# Impact of Diffusion Barriers to Small Cytotoxic Molecules on the Efficacy of Immunotherapy in Breast Cancer

**DOI:** 10.1371/journal.pone.0061398

**Published:** 2013-04-19

**Authors:** Hiranmoy Das, Zhihui Wang, M. Khalid Khan Niazi, Reeva Aggarwal, Jingwei Lu, Suman Kanji, Manjusri Das, Matthew Joseph, Metin Gurcan, Vittorio Cristini

**Affiliations:** 1 Department of Medicine, Wexner Medical Center, The Ohio State University, Columbus, Ohio, United States of America; 2 Department of Pathology, University of New Mexico, Albuquerque, New Mexico, United States of America; 3 Department of Biomedical Informatics, The Ohio State University, Columbus, Ohio, United States of America; 4 Department of Chemical Engineering and Center for Biomedical Engineering, University of New Mexico, Albuquerque, New Mexico, United States of America; Children’s Hospital Boston & Harvard Medical School, United States of America

## Abstract

Molecular-focused cancer therapies, e.g., molecularly targeted therapy and immunotherapy, so far demonstrate only limited efficacy in cancer patients. We hypothesize that underestimating the role of biophysical factors that impact the delivery of drugs or cytotoxic cells to the target sites (for associated preferential cytotoxicity or cell signaling modulation) may be responsible for the poor clinical outcome. Therefore, instead of focusing exclusively on the investigation of molecular mechanisms in cancer cells, convection-diffusion of cytotoxic molecules and migration of cancer-killing cells within tumor tissue should be taken into account to improve therapeutic effectiveness. To test this hypothesis, we have developed a mathematical model of the interstitial diffusion and uptake of small cytotoxic molecules secreted by T-cells, which is capable of predicting breast cancer growth inhibition as measured both *in vitro* and *in vivo*. Our analysis shows that diffusion barriers of cytotoxic molecules conspire with γδ T-cell scarcity in tissue to limit the inhibitory effects of γδ T-cells on cancer cells. This may increase the necessary ratios of γδ T-cells to cancer cells within tissue to unrealistic values for having an intended therapeutic effect, and decrease the effectiveness of the immunotherapeutic treatment.

## Introduction

Despite advances in both the diagnosis and management of early-stage disease, breast cancer remains the most frequently diagnosed cancer among women and leading cause of death in women worldwide [1]. Especially, due to the involvement of various cell types, breast cancer is a highly heterogeneous disease and the biology remains poorly understood. Current standard therapies for breast cancer, such as surgery, radiotherapy or hormonal blockade are usually only effective at achieving initial disease control. New forms of breast cancer therapy are much needed.

T cells play a significant role in the immunosurveillance and destruction of cancer cells. The tumoricidal potential of γδ T-cells was derived from the observation of preferential expansion and infiltration of γδ T-cells in various types of tumors [2]–[4]. However, since then limited success was obtained clinically in attempts to translate this knowledge into effective immunotherapies [5]. One of the major impediments is the infiltration of sufficient number of such cytotoxic cells within the tumor. Indeed, a very small number of γδ T cells were found within the breast tumor tissues in patients with various stages of disease [2], [6].

T cells expressing γ and δ T cell receptor (TCR) chains represent only a small subset (2%–5%) of the total T cell population. γδ T cells recognize antigens directly without any requirement for antigen processing and presentation or major histocompatibility complex (MHC) molecules [7], [8]. Of the two major subsets of human γδ T cells, Vγ2Vδ2 (also known as Vγ9Vδ2, collectively designated Vγ2) T cells predominate in the peripheral blood and respond to microbial infections by recognizing small nonpeptide molecules [9]–[11]. Significant efforts are underway to investigate the mechanistic basis of innate immune response to cancer in the context of γδ T-cells [9], [12], [13]. As a major mechanism of γδ T-cell-mediated growth control, interferon-γ (IFN-γ), a small cytotoxic molecule secreted by γδ T-cells, promotes cancer cell cycle arrest and apoptosis. We have reported that upon activation, human γδ T cells secret IFN-γ in a dose-dependent manner and this is correlated with the γδ T cell-mediated cytotoxicity [12], [14].

Spatial effects associated with tumor-induced angiogenesis and vascular flow [15], [16], drug delivery and tumor response [17]–[19], drug target prediction and validation [20], [21], and the heterogeneous tumor microenvironment [22]–[25] have been modeled mathematically in different contexts. Inspired by the often staggering difference in small cytotoxic molecule-based (e.g., IFN-γ) breast cancer inhibitory effects between *in vitro* (nearly complete response) and *in vivo* (only partial response), here we focus on modeling spatial effects that are present in tissue *in vivo* but virtually absent in cell monolayers *in vitro*, specifically the diffusion of IFN-γ through tumor tissue. Our hypothesis is that diffusion gradients in relative scarcity of T-cells are a major factor in limiting immunotherapy efficacy. We test this hypothesis by comparing the mathematical model predictions of tumor cell kill (or inhibition) with our *in vitro* and *in vivo* measurements of γδ T-cell-mediated breast cancer growth control, and demonstrate the model’s predictive accuracy.

## Results

We model the diffusion of IFN-γ released by γδ T-cells. The fraction of tumor kill (or inhibition) 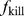 is thus predicted by the following equation (see **Methods**):
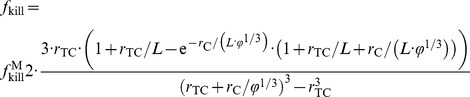
(1)


This equation relates 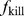 to the (larger) tumor kill fraction 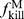 that would occur in an ideal experiment where all the cancer cells are exposed to a concentration of cytotoxic molecules equal to that produced by the T-cells (i.e., in the situation that no diffusion gradients are present); 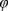 is the ratio of γδ T-cells to tumor cells; and 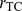 and 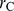 are the geometric mean diameters of γδ T-cells and tumor cells, respectively (rescaled with the diffusion penetration distance *L* of the small cytotoxic molecules, defined in Eq. (3) and (4)). One way to experimentally achieve 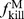 is to use a petri-dish where a monolayer of cancer cells is directly exposed to IFN-γ diluted in the serum. The condition of 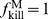 means that the concentration of cytotoxic molecules in the immediate vicinity of each T-cell is sufficient, if uniformly distributed in a medium, to kill all cancer cells in the petri-dish.

**Figure 1:**
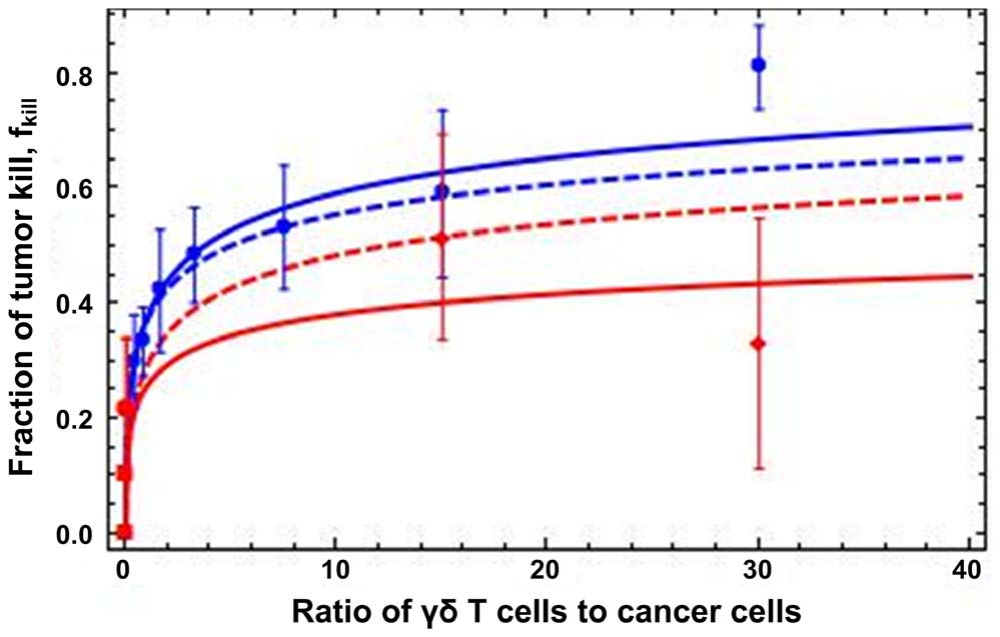
Fraction of tumor kill, 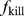, vs. ratio of γδ T-cells to cancer cells, 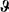. Experiments *in vitro* (blue circles with SD; n = 3) and *in vivo* (red diamonds: 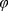 = 15, 30; red circles: 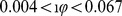; red squares: 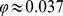; with SD; n = 3 or 4; some error bars not visible at the scale of the figure). Mathematical model Eq. (1) with 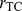 = 3 µm and 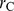 = 7 µm [1], [45] (blue solid curve: least-squares fit to the in-vitro data; 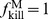, *L* = 36 µm; *R* ^2^ = 0.99 and *p*-value = 0.0002 for 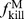 only; red solid curve: least-square fit to the in-vivo data; 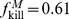, *L* = 679 µm; *R* ^2^ = 0.84 and *p*-value = 0.0037 for 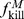 only; dashed blue curve: fit ignoring the outlier at 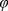 = 30; 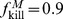 and 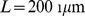; dashed red curve: fit ignoring the outlier at 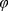 = 30; 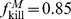 and *L* = 20µm). The fittings of the model, excluding the two outliers, are highly accurate in comparison with experimental data; in particular, the predicted diffusion penetration distance is much smaller *in vivo*.

**Figure 2:**
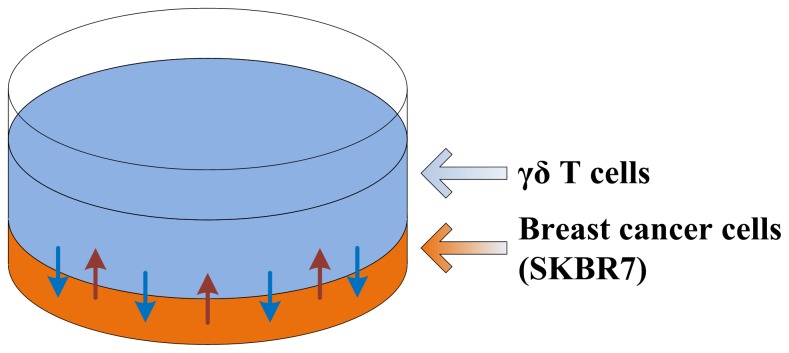
Schematic of the *in vitro* cell survivability assay. γδ T cells are plated on top of breast cancer cells.

We devised MTT cell viability and proliferation assays *in vitro* to investigate the effect of diffusion and T-cell scarcity under different ratios 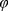 on the innate immune response of γδ T-cells. The T-cells used were expanded from peripheral blood of healthy human donors (∼99% CD3+, and ∼90% Vδ2+) [26] against the breast cancer cell line SKBR7 in a controlled environment. We found that the γδ T-cells inhibited breast cancer cell survival and proliferation in a dose-dependent manner after 24 h of co-culture (**Fig. 1**, blue circles). These results do not change at later times. Cancer cell inhibition increased as the ratio 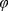 of γδ T-cells to cancer cells increased, as more cytotoxic small molecules were released into the serum and diffused through the cancer cell layer below the T-cells (see **Fig. 2** for an illustration of the *in vitro* experiments). The maximum cancer cell growth inhibition was observed at 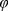 = 30. The mathematical model was calculated from Eq. (1) (**Fig. 1**, blue curves) and reproduces the *in vitro* data of cancer cell survival with accuracy. This confirms that diffusion gradients may play a role in reducing the effect of the treatment under T-cell scarcity, i.e., at smaller values of 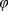. The outliers at 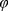 = 30 both in-vitro and in-vivo (red curves; see below) are discussed below in **Discussion**. Herein, the dashed curves represent the most accurate prediction as they do not include the two outliers.

To further investigate the relative importance of T-cell scarcity and diffusion gradients in limiting the efficacy of immunotherapy, we designed two *in-vivo* experimental approaches. First, we determined whether growth inhibitory effects of γδ T-cells observed *in vitro* translate to an ectopic xenograft model using NOD/SCID mice and co-injected (at time 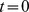) SKBR7 cells with γδ T-cells at two different ratios (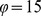 and 30; see **Methods**). We found that at *t* = 4 weeks, the tumor size was remarkably smaller in mice (51.6% of the control in average for 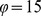 and 33.09% for 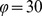) that received γδ T-cells compared to the case of using tumor cells alone (**Fig. 1**, red diamonds). Tumor formation was not detected in mice injected with higher doses of γδ T-cells alone (30 million cells/mouse), confirming that γδ T-cells do not have the potential to develop tumors.

**Figure 3:**
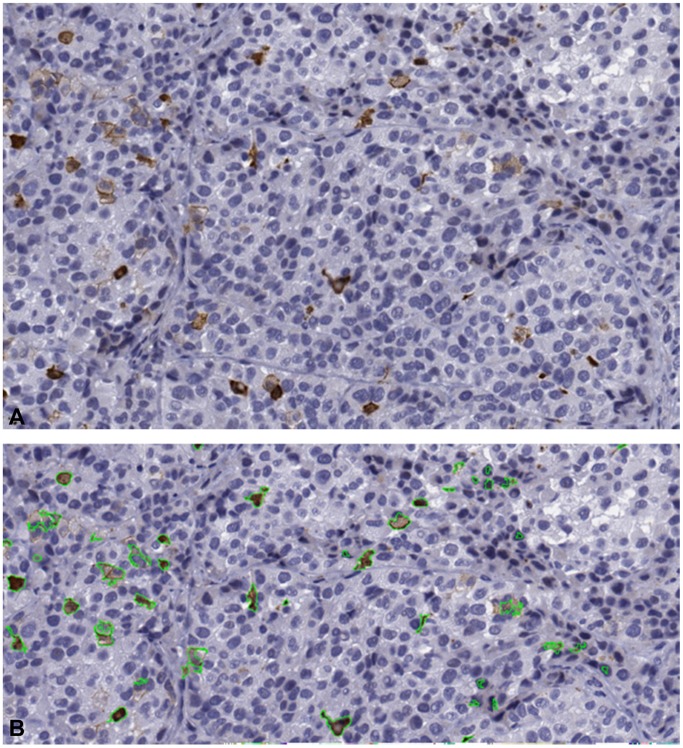
Automated quantitative determination of the amount of apoptosis in histological images. **A.** A sample high power field image stained with apoptosis marker, CC3, shown in brown hues. **B.** Segmentation of CC3 positive regions (outlined in green) in the image in **A**.

Using immunohistochemical techniques on the tissue obtained from the sacrificed mice after 4 weeks of injection, we found that animals that received γδ T-cells have a higher number of apoptotic cells (apoptosis marker CC3 processed with our image analysis algorithm; see **Fig. 3** and **Methods** for details). To quantify the amount of apoptosis in CC3 stained images, the following formulation was used:
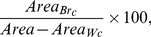
(2)where 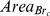 and 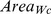 correspond to the number of pixels belonging to the brown (***Br_c_***) and white (***W_c_***) classes, respectively, while *Area* represents the total number of pixels in an image (see **Methods**). The average apoptotic index measured was 2–4% (**Fig. 1**, red squares) vs. 1% in the controls (i.e., no T-cells), thus demonstrating that T-cell-induced apoptosis contributed to the reduction in tumor growth. All values computed using Eq. (2) are reported in **Table 1** as they are difficult to see at the scale of **Fig. 1**. This was also confirmed by Western blot analysis (data not shown). Moreover, using human CD3 specific Ab (antibody) (see **Methods**), we confirmed a persisting small presence of γδ T-cells: 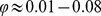 at *t* = 4 weeks (data not shown).

**Table 1:** Apoptosis calculation results.

Imaging Condition	Image 1 (%)	Image 2 (%)	Image 3 (%)
**SKBR Only**	0.84	1.41	1.79
**SKBR+1∶15 T**	2.26	4.67	2.50
**SKBR+1∶30 T**	2.18	1.84	1.68

The results in **Table 1** agree with the mathematical model predictions (**Fig. 1**, red solid curves obtained from Eq. (1) and fitted as described in caption). Cancer cells *in vitro* are layered on the plastic plate and relatively loosely packed (see **Fig. 2**); however, *in vivo* they make a solid mass of cells and become densely packed on each other, resulting in a smaller diffusion penetration distance *L*. The mathematical model demonstrates that diffusion barriers significantly hamper treatment effectiveness.

To confirm these conclusions, we performed a second set of experiments (see **Methods** for details) where we injected the γδ T-cells in the tail-vein at *t* = 2 weeks, when the tumors were already established (one million tumor cells were injected into the mouse initially, and were allowed to grow for two weeks). After injecting 100 million γδ T-cells, tumors were allowed to grow for four additional weeks. The tumor volume was then assessed at *t* = 6 weeks. In the absence of direct measurement, we calculated the fraction of γδ T-cells in the tumors by assuming a tumor tissue density equal to water and exponential growth of the tumor; thus, from the tumor control size of ca. 285 million cells at *t* = 4 weeks, we estimated that there were ca. 17 million tumor cells at *t* = 2 weeks (when the tail-injection of γδ T-cells occurred). Considering that the tumor mass was 0.285 g whereas the weight of the mouse was 25 g, the above reasoning led to the estimate: 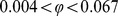. As expected, under the conditions of smaller 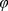 values, less (ca. 22.1%) tumor growth inhibition was achieved (**Fig. 1**, red circles). In fact, with the current cancer treatment methods, the ratio of γδ T-cells to cancer cells is likely to be small. The mathematical model Eq. (1) satisfactorily agrees with this data.

## Discussion

Increasing evidence for cytotoxic antitumor activities of human Vγ2Vδ2 T cells against a large range of tumor types suggests that human γδ T cells are being considered as one of the major effector cells for immunotherapy (reviewed in [12], [14], [27]). Activated Vγ2Vδ2 T cells only provide tumor-targeted recognition and efficient killing of tumor cells. Immune effector cells recognize and destroy tumor targets via a number of mechanisms including death receptor/ligands interactions with TRAIL and FasL, recognition of stress-inducible molecules by NKG2D, and by release of perforin/granzymes or cytokines such as IFN-γ. One or more of these pathways may be involved in the synergy during the activation of Vγ2Vδ2 T cells. Moreover, Vγ2Vδ2 T cells employ different cytotoxic mechanisms depending on the mode of target cell recognition [28]. Previous studies have demonstrated the importance of NKG2D-MICA/B interactions for tumor cell recognition and effective cytotoxic activity by γδ T cells [12], [29], in addition to perforin/granzyme-dominated killing [28]. Increased release of stored perforin by Vγ2Vδ2 T cells with exposure to activation was also reported [20]. Other immune cells, such as monocytes, macrophage, and dendritic cells may infiltrate into the tumor and play an important role in antigen presentation to the cytotoxic T cells for their activation and enhanced cytotoxic lysis [28]. Recent reports indicate that subtype of macrophage (M1 and M2) play opposite role in tumor suppression (M1) and tumor promotion (M2) [29], depending on their number could modulate fate of the tumor.

IFN-γ is produced and secreted upon activation of Vγ2Vδ2 T cells, and represents one of the major pathways for cytotoxic lysis of various tumor cells. The production of IFN-γ was found to be substantially higher after exposure to activating molecules, but have no correlation with the degree of Vγ2Vδ2 T cell-mediated cytotoxicity [20]. A previous study also showed that cell-cell contact was essential for Vγ2Vδ2 T cells cytotoxic activity, but soluble factors such as IFN-γ were not directly involved despite high levels of production [21]. We hypothesize that the diffusion process is a critical determinant for cytotoxic lysis of tumors by γδ T cells.

How to overcome these diffusion barriers to transport a sufficient amount of γδ T cells to the tumor? One may consider increasing the volume of γδ T cells, but this may potentially introduce undesirable adverse effects due to the increased toxicity [30]. Here, the concept of combination therapy may help, but it is important to identify specific biophysical transport barriers first (e.g., diffusion gradients of substrates (oxygen, glucose, etc.) and abnormal tumor vascularization). With this in mind, we can, for example, normalize tumor vasculature through anti-VEGF antibody (e.g., Avastin) [31], so more γδ T cells can reach the tumor. We can also use the drug-encapsulated nanoparticle-based approach [32] to enhance the delivery of γδ T cells. However, all these forms of combination therapy are subject to further investigation. Finally, γδ T cells are extremely sensitive to activation-induced cell death and rapidly undergo apoptosis upon pharmacologic or even physiologic stimulation [33]–[35], and thus isolating or expanding viable, functional human γδ T cells is an obstacle. Fortunately, we and others have made progress in expanding γδ T cells for the large-scale application without compromising their characteristics [26], which enables us to examine the possibility of developing γδ T cell-based treatment methods into clinical use.

The *in-vivo* co-injection values of 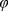 = 15, 30 at *t* = 0 (**Fig. 1**) would be highly unrealistic to achieve in patients, while the much smaller values of 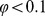 measured at *t = *2−4 weeks are likely more reflective of those achievable in the clinic, thus revealing how profoundly diffusion barriers would hamper effectiveness of clinical application. This result also partly explains why current γδ T cell-based therapies fail when applied to clinical practice, while their effects on cancer growth control and inhibition have been proven in lab-based experiments. Overall, this study strongly suggests that, in order to develop more effective γδ T cell based immunotherapies, the effect of diffusion barriers on delivering γδ T cells and their released cytotoxic molecules (such as, IFN-γ) to tumor cells should be taken into account, in addition to exploring the underlying molecular mechanisms that regulate cancer cell behavior.

Eq. (1) was derived by assuming that T-cells are scarce enough that, at any point in space, the concentration of IFN-γ is well approximated assuming the presence of only one T-cell source. The last experimental points in **Fig. 1** (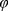 = 30) do not agree with the curves obtained from the mathematical model (dashed). *In vitro*, this was expected since at those high ratios 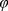, the actual concentration of small cytotoxic molecules, to which the cancer cells are exposed, is higher than predicted by the “scarce T-cell” model due to the proximity of many T-cells to each cancer cell. On the other hand, *less* cell inhibition observed *in vivo* at 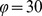 might be explained by the fact that γδ T-cells express both Fas and FasL molecules on their surface (Fas/FasL interaction has a central role in the regulation of programmed cell death [36]). When higher amounts of cells are activated, they may kill each other via Fas/FasL interaction mechanism before killing cancer cells [37]. Alternatively, injection of 30 million T-cells and 1 million cancer cells may put extra stress on the injected cells because of limited nutrients and oxygen supply. While the majority of cells die, it is reasonable to assume that γδ T-cells die faster as they are primary cultured cells, rather than cancer cells, which are adapted to survive in hypoxic conditions.

γδ T cells can be treated as chemotherapy drugs (while γδ T cells are live and more active), and thus with appropriate modifications, the general modeling method (Eq. (1)) can be used for studying tumor drug response to correlate tumor microenvironment to chemotherapy effectiveness. For instance, we have recently developed a model of mass transport using the same method presented herein to study the diffusion of chemotherapy drugs in colorectal cancer (CRC) metastases in the human liver. The model accurately predicts the fraction of dead tumor cells due to treatment, and agrees well with the patient samples (data not shown).

The amount of apoptosis was calculated automatically using image analysis algorithms. These algorithms provide the consistency needed for the accurate measurement of parameters needed for mathematical modeling. In case of human readers, there is a large intra- and inter-reader variability, so a model depending on the readers’ subjective determination may fail to explain the underlying mechanism. The challenge when developing image analysis algorithms is to account for the differences in the images of the immunohistochemically stained slides, that are caused by inevitable differences in slice thickness and the amount of stain used between different labs, even within the same lab or same slide. Here, we have successfully developed algorithms that are adaptive to these changes (see **Image Analysis**).

In summary, our finding explained, from a mathematical perspective, why current lysis of tumor by γδ T-cells *in vivo* is not as effective as that *in vitro*. Using the model, we predicted that the diffusion process plays a critical role in the γδ T-cell mediated cytotoxic lysis of the tumor cells. If the diffusion barrier issues can be solved someway, cytotoxic lysis can be enhanced and a cytotoxicity level sufficient for killing, or inhibiting the growth of, cancer cells might be achieved with the current γδ T-cell based immunotherapy.

## Methods

### Mathematical Model

**Figure 4:**
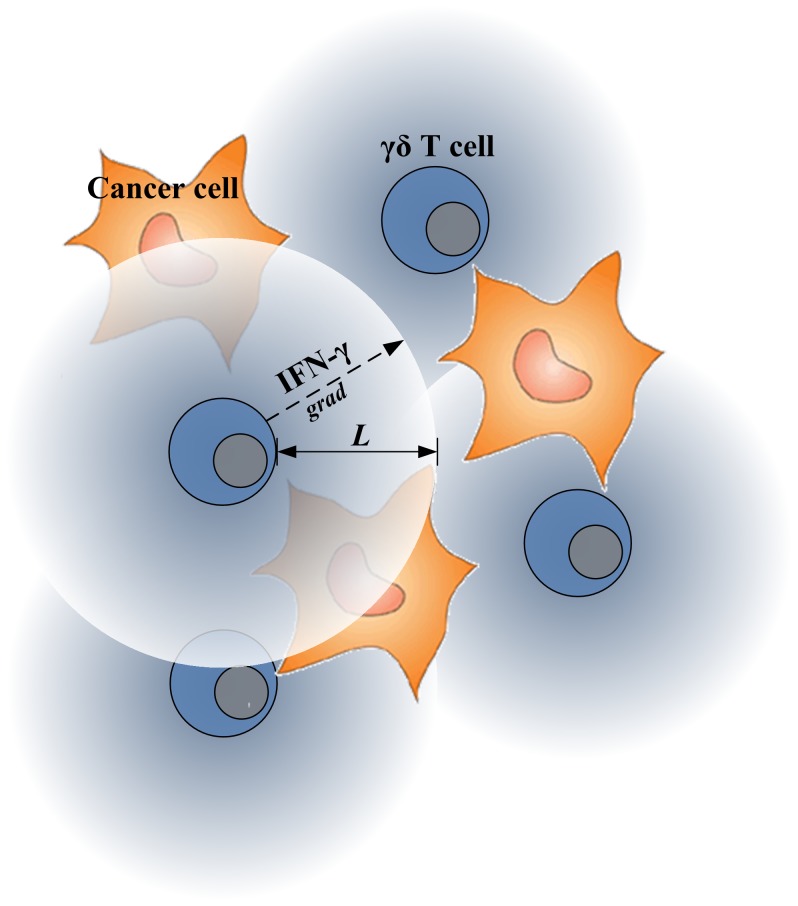
Schematic of interaction of γδ T cells and cancer cells through IFN-γ.

IFN-γ, released by γδ T cells, induces antiproliferative and proapoptotic effects on many types of cancer cells through a rather complex, interconnected signaling mechanism [38]. Here, we simplified this complex IFN-γ-mediated cancer cell growth inhibition system, by lumping the binding process of IFN-γ to its receptor with all subsequent signaling processes into a single phenomenological parameter, i.e., the IFN-γ uptake rate. We also assumed that the processes of diffusion and uptake of IFN-γ over time across the tissue region where γδ T cells and cancer cells co-exist are quasi-steady compared to the proliferation and death processes. **Figure 4** shows a schematic of how γδ T cells interact with cancer cells through small cytotoxic molecules, IFN-γ. It is intuitive to find that biophysical properties of IFN-γ, such as diffusion penetration length (*L*), may limit the effects of IFN-γ on inhibiting cancer cell growth. For example, if *L* is too small and γδ T cells are scare, IFN-γ may not be able to reach and bind to its receptors expressed on the surface of tumor cells.

The concentration of IFN-γ across the tissue region (a mixture of γδ T cells and cancer cells) is described using the following partial differential equation:
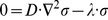
(3)where σ represents the interstitial concentration of IFN-γ, *λ* is the uptake rate of IFN-γ by the cancer cells, and *D* is the interstitial diffusion constant of IFN-γ. The diffusion penetration distance of IFN-γ is then:



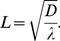
(4)Thus, Eq. (3) models diffusion of IFN-γ with penetration distance *L*. Note that *L* was estimated by comparing with doxorubicin’s values [18] according to the following equation:
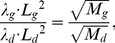
(5)where *λ_d_*, *L_d_*, and *M_d_* are uptake rate, penetration length, and molecule weight for doxorubicin, and *λ_g_*, *L_g_*, and *M_g_* are for IFN-γ, respectively. We find the decaying solution:
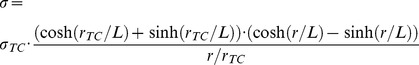
(6)which satisfies the boundary condition 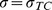 at the T-cell membrane (of equivalent radius 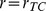). Note that this solution is not correct in the limit of infinite diffusion penetration (i.e., no uptake: 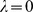). We choose this solution because it decays away from the T-cell; whereas the solution 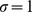 that correctly describes 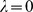 conditions can be obtained without imposing that the general solution of Eq. (3) decays away from the T-cell. This, however, leaves one boundary condition undetermined and is the subject of studies underway.

Assuming that the diffusion penetration distance is small compared to the inter T-cell distance, we can ignore the overlap of the diffusion fields around multiple T-cells, and thus define the fraction of tumor kill by integrating Eq. (6) across a spherical volume 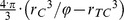 surrounding a T-cell and with radius extending to a distance set by the fraction 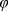 of T-cells to tumor cells (of radius 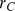):
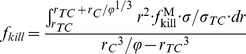
(7)which leads to Eq. (1).

### Experiments

#### Isolation and *in-vitro* culture of γδ T cells

Human peripheral blood (30 ml) was collected from healthy adult donors after obtaining the IRB approval from Wexner Medical Center at The Ohio State University and written consents from donors. Freshly collected blood was processed to isolate peripheral blood mononuclear cells (PBMC) following previously published protocols [12], [39], [40]. In brief, the peripheral blood was diluted twice with phosphate buffer saline (PBS, pH 7.4) and carefully layered over 10 ml of Ficoll-Paque Plus solution (GE Healthcare, Uppsala, Sweden). After 30 minutes of centrifugation in a swinging bucket rotor at 1400 rpm at room temp (24°C), the upper layer was aspirated out and the mononuclear cell layer (buffy coat) was collected. The buffy coat was washed three times with PBS to remove platelets. One million PBMC in each well were stimulated with 10 µM risedronate in a 24-well plate using 1 ml RPMI 1640 supplemented with 10% fetal bovine serum (FBS, HyClone Lab Inc, Logan, UT), 2 mM glutamine, 1 nM β-mercapto ethanol, 1 nM HEPES and 100 IU of penicillin and streptomycin at 37°C incubator. Recombinant IL-2 (rIL-2), 0.5 nM (PeproTech Inc. Rocky Hill, NJ) was added to the culture on days 3 and 7. Cells were split after day 10 using the complete RPMI 1640 media supplemented with 0.5 nM rIL-2. Flowcytometric (FACS) analysis was performed at day 14, to evaluate phenotype of the expanded cell. Cells were used between 15–19 days of initial culture for further experiments discussed below.

#### Xenografts of the tumor cells

Immunocompromised (NOD/SCID) male mice (8 weeks old) were categorized into four groups. Each NOD/SCID mouse was subcutaneously injected with either tumor cells (SKBR7; one million) or γδ T cells (15 million) separately or co-injected, i.e., SKBR7 plus γδ T cells, at a ratio of 1∶15 or 1∶30. Breast tumor cells were plated and cultured in 10 cm cell culture sterile petri dishes, three days before the injection. After three days, cells were trypsinized and counted. The cells were washed with 1 × PBS to get rid of the cell culture medium and diluted at the concentration of 1 million cells per 200 µl (1 × PBS). Similarly, on day 17 of culture, γδ T cells were isolated, washed and diluted at the concentration of either 15 million or 30 million per 200 µl (1 × PBS) and injected. After four weeks of injection, tumors were harvested and weighed, divided into 2 equal halves for further experiments. One half was formalin fixed for histology, immunohistochemistry or frozen at −80 C for later use or used for isolation of total protein.

#### Immunohistochemistry of tumor tissues

The harvested tissues were formalin fixed, paraffin-blocked, sectioned and immuno labeled with cleaved caspase-3 or anti-CD3 primary antibody and stained using horse peroxidase conjugated DAB staining by pathology core facility personnel at College of Veterinary Medicine, the Ohio State University.

#### Image database

Four different conditions were analyzed (negative control, SKBR only, SKBR+T15, and SKBR+T30). The tissue samples were obtained in accordance with the approval of Institutional Animal Care and Use Committee (IACUC), The Ohio State University Office of Research Integrity, protocol **#** 2010A00000139, dated 8/18/2010 and renewed annually). Each tissue sample was cut at a thickness of 4 µm and stained by Cleaved Caspase 3 (CC3) staining. Each stained slide was digitized using a ScanScope T2 digitizer (Aperio, San Diego, CA) at 40× magnifications. Three representative areas of size equivalent to one high power field (hpf, approximately 0.159 µm^2^) were randomly selected by a blinded investigator, resulting in 12 images corresponding to the four different conditions.

### Image Analysis

The objective of image analysis is to quantitatively determine the amount of apoptosis, which is manifested as brown hues in CC3 stains. A typical CC3 image generally contains three major hue classes: white (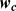), blue (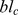), and brown (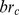) (see **Fig. 3A**). Based on visual inspection, it can be thought that these hue classes can be easily separated by using a simple unsupervised clustering method such as k-means clustering which assigns pixels to each hue class (i.e. 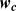, 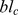, and 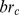) based on Voronic cell of its centroids [41]. However, due to large variability in shades of blue and brown pixels, k-means often results in misclassification. Consequently, the 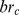 class obtained using k-means often contains the mixture of brown and blue pixels. To properly segment the brown and the blue pixels present in the 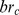 class (obtained using k-means), we propose a linear transformation in the 1976 color space [42], which translates this oversegmentation problem into a simple threshold problem.

In the presence of shades of blue and brown pixels, the 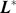 channel in the color space correlates quite strongly (on average, the cross-correlation value is greater than 0.7) with the 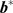 channel of the image. It can also be observed that, the regions corresponding to the brown cells in the 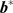 channel have slightly higher intensity values (average intensity values corresponding to the blue, white, and brown regions in 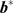 channel are 105, 120, and 124, respectively) as compared to those of blue cells and the background. The difference among the average intensity values corresponding to blue, white and brown regions is so marginal that the 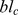 and 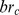 classes are indistinguishable in the probability mass function of the 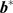 channel. In the 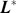 channel, the area corresponding to the 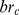 has lower intensity values than those of 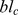 regions with an average intensity difference of 10. However, there is no single threshold to properly separate the 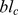 and 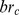 classes from the probability mass function of the 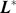 channel. As the 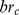 class changes its intensity values from being highest in 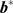 to lowest in the 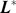 channel, we can conclude that 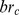 class corresponds to the source of information (entropy) between 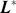 and 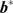 channel. As green and magenta are equally likely to be present in different shades of brown and blue except for the white background, the 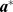 channel does not contain much discernable information. For this reason, its probability mass function tends to be normally distributed. Although the 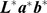 color space provides perceptual uniformity in comparison to other color spaces, its color (i.e., 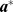, 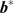) and luminance (i.e., 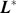) decompositions do not correspond to the information based visual decomposition of a CC3 stained image (i.e. meaningful segmentation into biologically relevant components). Therefore, standard perceptually uniform color spaces (e.g., the ) do not provide information based visual decomposition of the image. To achieve the *information based visual decomposition* of an image, it is often desirable to de-correlate the individual channels by virtue of some transformation.

Principle component analysis (PCA) is one of the most widely used linear transformations where the eigenvectors serve as the new orthogonal coordinate system [43]. PCA rotates the current axis so that the eigenvector corresponding to the highest eigenvalue points in the direction of maximum information content in an image. In our case, the application of PCA will transform the naive basis vectors of the 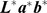 color space to a set of new orthogonal basis vectors. The projection of the 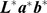 image onto these new basis vectors will provide much more meaningful insight into the biological information content presented in an image. The projected image ***P*** for an image 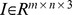 in 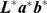 color space can be computed as:
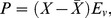
(8)where 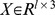 (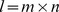) represents the rearranged version of *I*, while 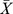 correspond to the average of *X* along rows. 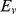 is a matrix whose columns are eigenvectors of the covariance matrix *S*:
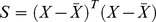
(9)where *T* stands for matrix transpose. In Eq. (8) and (9), rows of *X* correspond to the pixels in *I* while the three columns of *X* represent the intensity values in 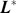, 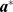, and 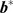 channel of *I*. It is worth mentioning that this rearrangement of pixels from *I* to *X* is necessary to simplify the computation of ***P***.

As we are just changing the coordinate system, the projected image ***P***, after PCA transformation, will consist of the same number of channels as the original image ***I***. It is expected that the first channel in ***P*** will directly correspond to the 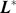 channel of image ***I***, as 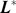 channel in ***I*** is much richer in information content as compared to the other two channels. The second channel in the ***P*** image will highlight the region corresponding to the 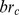 class because this class was the source of entropy (information) between the 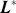 and 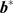 channels in ***I***. The third channel generally corresponds to noise and is ignored.

To properly segment the brown and the blue pixels in the 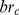 class, the corresponding pixels (pixels belonging to the 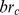 class obtained using k-means) in the second channel of ***P*** are subjected to entropy based histogram threshold [44]. The threshold algorithm divides the histogram of the image into two probability distributions, one representing the brown pixels and the other corresponding to the blue pixels. The algorithm chooses a threshold ***t*** such that the sum of the entropies of these probability distributions is maximized. More precisely, the value of *j* which maximizes the following objective function is set to ***t***.



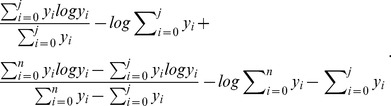
(10)


Here, *y_i_* represents the number of pixels with intensity value *i*, while *n* represents the highest intensity value from the set of pixels under consideration. The resulting binary image is post-processed with morphological operations to fill small holes and to remove the small isolated components. **Figure 3B** shows a sample output corresponding to the image in **Fig. 3A**.
