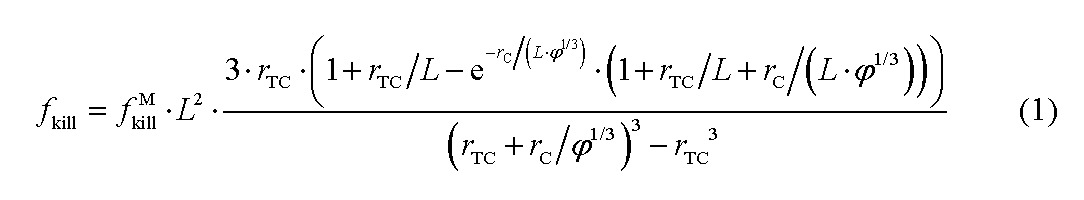# Correction: Impact of Diffusion Barriers to Small Cytotoxic Molecules on the Efficacy of Immunotherapy in Breast Cancer

**DOI:** 10.1371/annotation/94eab1ba-657a-445d-abf2-a709fc6a9806

**Published:** 2013-10-25

**Authors:** Hiranmoy Das, Zhihui Wang, M. Khalid Khan Niazi, Reeva Aggarwal, Jingwei Lu, Suman Kanji, Manjusri Das, Matthew Joseph, Metin Gurcan, Vittorio Cristini

In the Results section, there is an error in the first equation. Please view the complete, correct equation here: